# Supplementary material for: Genome-scale identification of transcription factors that mediate an inflammatory network during breast cellular transformation
Source: Nat Commun. 2018 May 25;9:2068. doi: 10.1038/s41467-018-04406-2 (PMC5970197; doi:10.1038/s41467-018-04406-2)
Supplement: Supplementary file 1 — Supplementary Information [file 41467_2018_4406_MOESM1_ESM.pdf]

Supplementary Information

Genome-scale identification of transcription factors that mediate an inflammatory network during breast cellular transformation

Ji et al

GO ID	GO Name	MAX	Continually upregulated	Early upregulated	Intermediate upregulated	Continually downregulated
GO:0007167	enzyme linked receptor protein signaling pathway	5.26	0.05	5.26	0.03	1.13
GO:0042981	regulation of apoptosis	5.13	1.11	5.13	0.44	2.59
GO:0001944	vasculature development	4.86	1.18	4.86	0.23	0.52
GO:0048514	blood vessel morphogenesis	4.8	0.75	4.8	0.16	0.24
GO:0009611	response to wounding	4.76	4.76	2.04	1.63	0.07
GO:0010604	positive regulation of macromolecule metabolic process	3.78	0.06	3.78	0.44	0.65
GO:0051094	positive regulation of developmental process	3.59	0.13	3.59	0.85	0.00
GO:0001525	angiogenesis	3.39	0.66	3.39	0.10	0.00
GO:0034097	response to cytokine stimulus	3.33	3.33	0.05	0.00	0.01
GO:0042127	regulation of cell proliferation	3.05	1.05	2.32	0.26	3.05
GO:0030334	regulation of cell migration	2.92	0.00	2.92	0.23	1.02
GO:0042325	regulation of phosphorylation	2.91	0.00	2.91	0.21	0.53
GO:0051674	localization of cell	2.59	0.39	0.72	0.31	2.59
GO:0007155	cell adhesion	1.97	0.2	1.97	0.6	0.06
GO:0051726	regulation of cell cycle	1.89	0.01	1.89	0.13	0.96
GO:0006954	inflammatory response	1.51	1.51	0.37	0.96	0.01

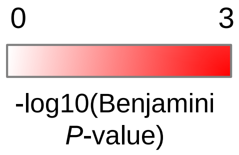

Supplementary Figure 1. Gene ontology of differential expressed gene clusters

The -log10(Benjamini *P*-value) are shown. The pathways were sorted based on the maximum -log10(Benjamini *P*-value) in 4 differential expressed gene clusters.

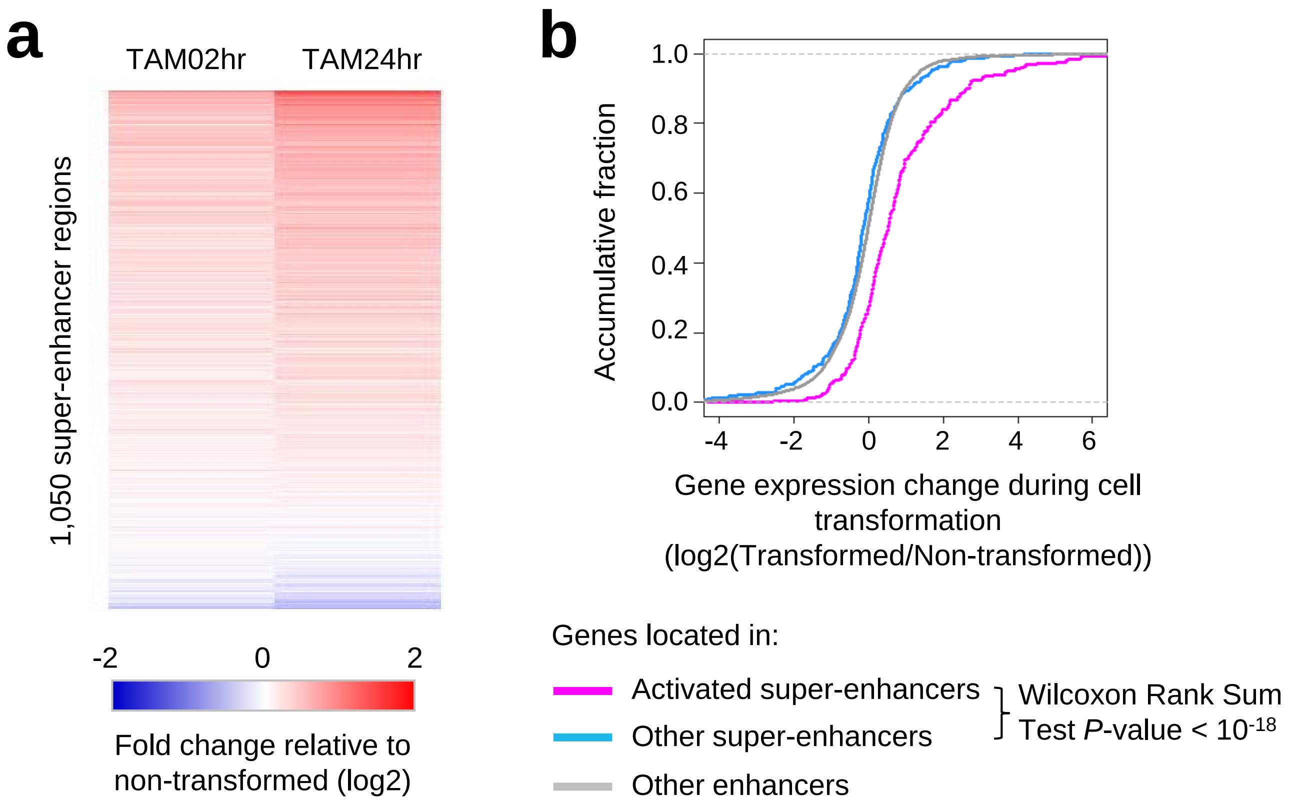

Supplementary Figure 2. Analyses of superenhancers during transformation

a 1,050 superenhancers were identified in at least one time point during MCF10A-ER-Src cell transformation. The heatmap of fold changes of acetylation level relative to TAM00hr was shown. **b** Comparing differential expression of superenhancer genes and other genes during the cell transformation. Activated super-enhancers were those showing >1.5 fold of increased acetylation levels.

GO ID	GO term	Activated Superenhancers	Other Superenhancers
GO:0051270	regulation of cell motion	2	10.33
GO:0042127	regulation of cell proliferation	1.94	6.37
GO:0001568	blood vessel development	3.19	3.72
GO:0007242	intracellular signaling cascade	3.71	1.6
GO:0043067	regulation of programmed cell death	1.64	3.71
GO:0042981	regulation of apoptosis	1.67	3.7
GO:0001525	angiogenesis	2.29	3.64
GO:0006357	regulation of transcription from RNA polymerase II promoter	1.87	3.19
GO:0010604	positive regulation of macromolecule metabolic process	1.21	2.69
GO:0009611	response to wounding	2.61	2.37
GO:0010033	response to organic substance	2.54	1.53
GO:0042060	wound healing	0.96	2.38
GO:0030035	microspike assembly	0	2.37
GO:0007167	enzyme linked receptor protein signaling pathway	0.28	2.16
GO:0007243	protein kinase cascade	2.04	0.63
GO:0022604	regulation of cell morphogenesis	0.9	2.04
GO:0009991	response to extracellular stimulus	1.94	0.66
GO:0006796	phosphate metabolic process	1.85	1.76

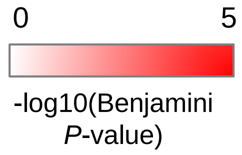

Supplementary Figure 3. Gene ontology of genes in super-enhancer regions
Activated super-enhancers were those showing >1.5 fold of increased acetylation levels.

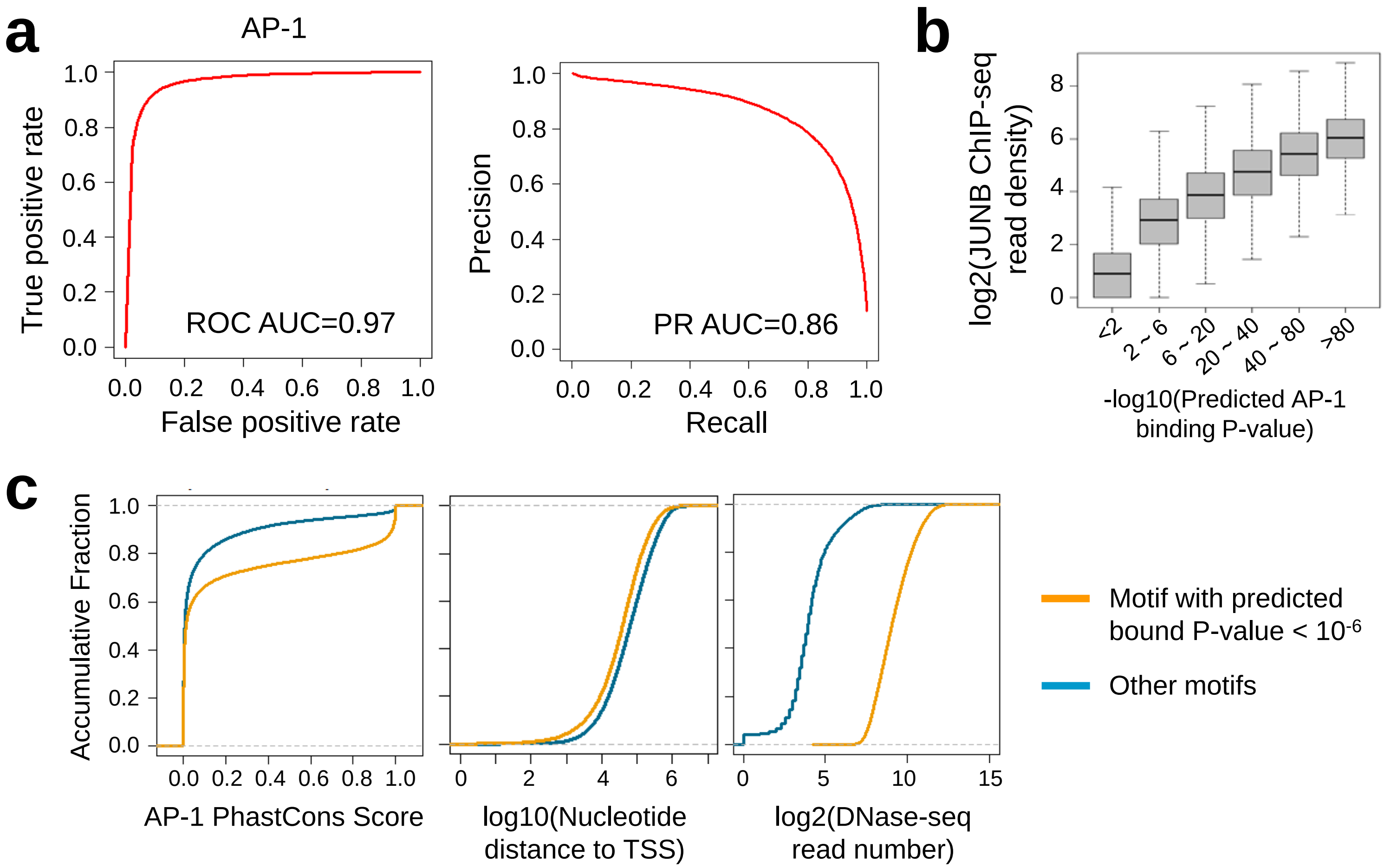

Supplementary Figure 4. Performance of AccessTF to identify AP-1 binding sites

a Area under ROC curves (ROC AUC) and Area under Precision-Recall Curves (PR AUC) for measuring the performance of AccessTF predicting the binding status of AP-1. **b** The AP-1 motifs were grouped based on predicted binding probabilities. And JUNB binding levels in 400 nt region around the motifs estimated by ChIP-seq data after cell transformation, were plotted. **c** Comparing features of AP-1 motifs with predicted bound P-value < 10^{-6} versus others.

Factor	Motif	ROC AUC	PR AUC
AP1	MA0099.2	0.96	0.82
GATA2	MA0036.2	0.99	0.78
ELK1	MA0028.1	0.99	0.86
CTCF	MA0139.1	0.90	0.75
IRF1	MA0050.2	0.87	0.61
CEBPB	MA0466.1	0.77	0.40
E2F4	MA0470.1	0.96	0.81
NF-YA	MA0060.2	0.92	0.60

Supplementary Figure 5. Performance of AccessTF in K562 cells
Area under ROC Curve (ROC AUC) and Area under Precision-recall Curve (PR-AUC) values measuring the performance of AccessTF predicting the binding status of indicated transcription factors in K562 cells.

a

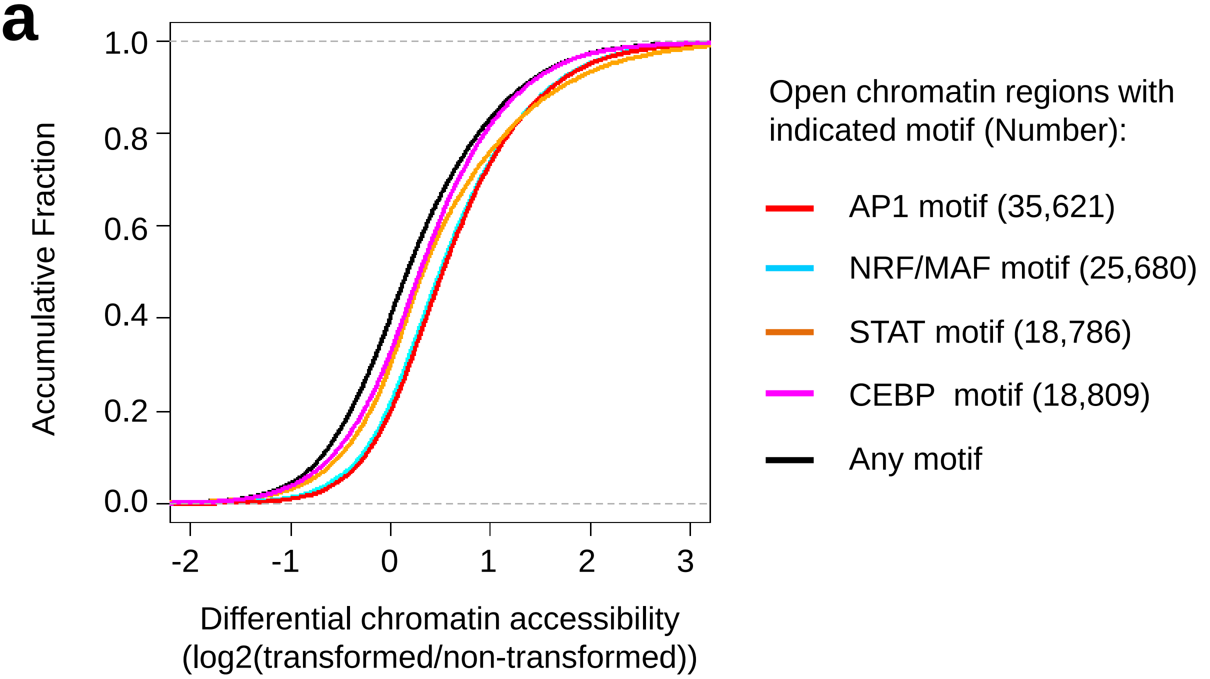

b

	Continuously upregulated	Early upregulated	Intermediate upregulated	Continually downregulated
AP1	23.9	16.0	6.7	20.2
NFE/MAF	25.9	16.2	6.2	17.9
STAT	24.0	16.1	8.0	15.5
CEBP	28.9	16.1	9.1	20.2

-log(*P*-value)

0 30

c

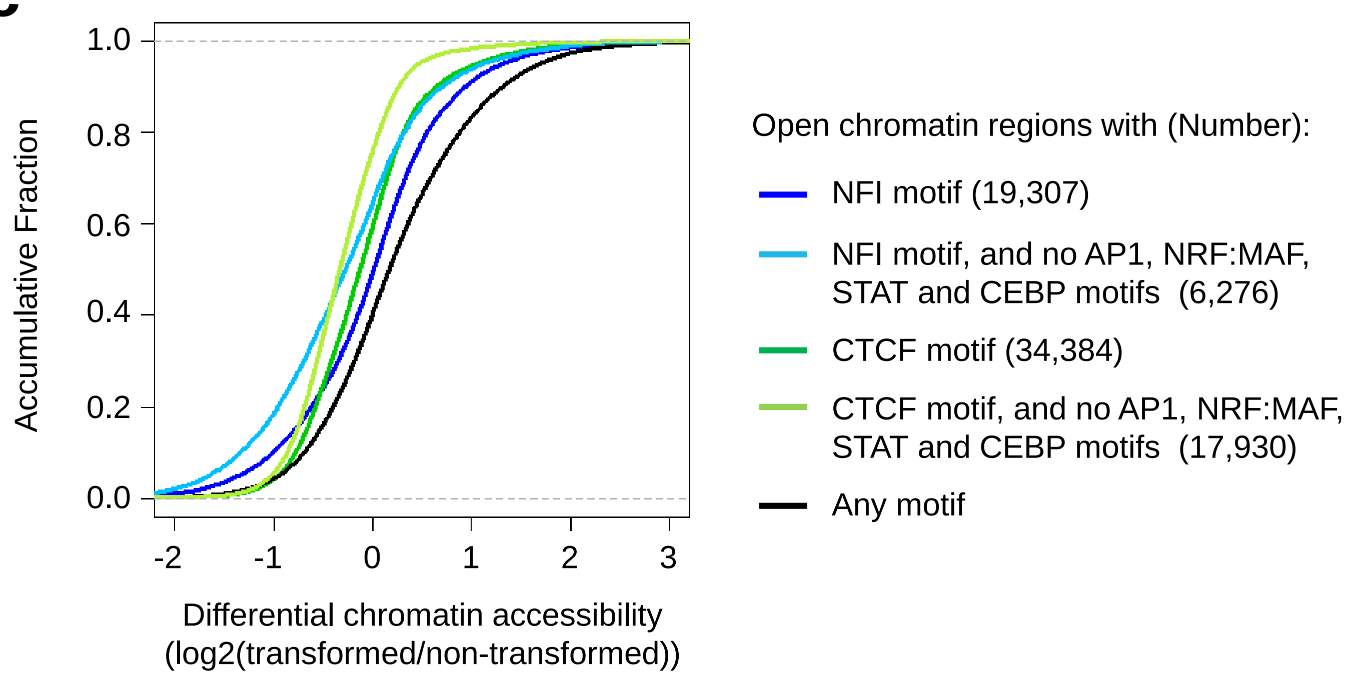

Supplementary Figure 6. Motif enrichment in dynamically accessible chromatin regions
a Comparing open chromatin regions with indicated factor binding motifs learned from AccessTF versus all regions. The fold-change chromatin accessibility during transformation are shown. **b** Relative enrichment of motifs in promoters/enhancers of differentially expressed gene clusters as compared to genes that do not show significant differential expression. **c** Comparing open chromatin regions with indicated factor binding motifs versus all regions. The fold-change of chromatin accessibility during the transformation are shown.

Figure S7

GO ID	GO term	Benjamini *P*-value
GO:0000278	mitotic cell cycle	6.12E-16
GO:0006412	translation	5.65E-15
GO:0007049	cell cycle	4.85E-13
GO:0006396	RNA processing	3.09E-12
GO:0000280	nuclear division	2.27E-11
GO:0048285	organelle fission	1.03E-10
GO:0034621	cellular macromolecular complex subunit organization	3.41E-09
GO:0006259	DNA metabolic process	1.79E-08
GO:0051276	chromosome organization	3.34E-08
GO:0022613	ribonucleoprotein complex biogenesis	3.88E-08
GO:0044265	cellular macromolecule catabolic process	1.73E-07
GO:0030163	protein catabolic process	2.01E-07
GO:0065003	macromolecular complex assembly	2.04E-07
GO:0043632	modification-dependent macromolecule catabolic process	2.55E-07
GO:0006260	DNA replication	4.53E-07
GO:0009057	macromolecule catabolic process	6.47E-07
GO:0051603	proteolysis involved in cellular protein catabolic process	7.43E-07
GO:0031497	chromatin assembly	8.76E-07

Supplementary Figure 7. Gene ontology of downregulated genes
Gene ontology analyses of 1,326 genes downregulated upon 6 factor knockdowns and not upregulated during cell transformation. The -log10(Benjamini *P*-value) are shown.
