# Supplementary material for: Genome-scale identification of transcription factors that mediate an inflammatory network during breast cellular transformation
Source: Nat Commun. 2018 May 25;9:2068. doi: 10.1038/s41467-018-04406-2 (PMC5970197; doi:10.1038/s41467-018-04406-2)
Supplement: Supplementary file 3 — Description of Additional Supplementary Files [file 41467_2018_4406_MOESM3_ESM.pdf]

Description of Additional Supplementary Files

File Name: Supplementary Data 1
Description: List of superenhancers identified using H3K27ac ChIP-seq data during cell transformation.

File Name: Supplementary Data 2
Description: List of transcription factors ranked based on TFScores.

File Name: Supplementary Data 3
Description: siRNAs for transcription factors in Figure 5.

File Name: Supplementary Data 4
Description: qPCR Primer sequences for transcription factors in Figure 5.
